# High referral rates to secondary care by general practitioners in Norway are associated with GPs’ gender and specialist qualifications in family medicine, a study of 4350 consultations

**DOI:** 10.1186/1472-6963-13-147

**Published:** 2013-04-23

**Authors:** Unni Ringberg, Nils Fleten, Trygve S Deraas, Toralf Hasvold, OlavHelge Førde

**Affiliations:** 1 Faculty of Health Sciences, Department of Community Medicine, University of Tromsø, Tromsø, Norway; 2 Centre of Clinical Documentation and Evaluation, Northern Norway Regional Health Authority, Tromsø, Norway

## Abstract

**Background:**

Referral rates of general practitioners (GPs) are an important determinant of secondary care utilization. The variation in these rates across GPs is considerable, and cannot be explained by patient morbidity alone. The main objective of this study was to assess the GPs’ referral rate to secondary care in Norway, any associations between the referral decision and patient, GP, health care characteristics and who initiated the referring issue in the consultation.

**Methods:**

The probabilities of referral to secondary care and/or radiological examination were examined in 100 consecutive consultations of 44 randomly chosen Norwegian GPs. The GPs recorded whether the issue of referral was introduced, who introduced it and if the patient was referred. Multilevel and naive multivariable logistic regression analyses were performed to explore associations between the probability of referral and patient, GP and health care characteristics.

**Results:**

Of the 4350 consultations included, 13.7% (GP range 4.0%-28.0%) of patients were referred to secondary somatic and psychiatric care. Female GPs referred significantly more frequently than male GPs (16.0% versus 12.6%, adjusted odds ratio, AOR, 1.25), specialists in family medicine less frequently than their counterparts (12.5% versus 14.9%, AOR 0.76) and salaried GPs more frequently than private practitioners (16.2% versus 12.1%, AOR 1.36).

In 4.2% (GP range 0%-12.9%) of the consultations, patients were referred to radiological examination. Specialists in family medicine, salaried GPs and GPs with a Norwegian medical degree referred significantly more frequently to radiological examination than their counterparts (AOR 1.93, 2.00 and 1.73, respectively).

The issue of referral was introduced in 23% of the consultations, and in 70.6% of these cases by the GP. The high referrers introduced the referral issue significantly more frequently and also referred a significantly larger proportion when the issue was introduced.

**Conclusions:**

The main finding of the present study was a high overall referral rate, and a striking range among the GPs. Male GPs and specialists in family medicine referred significantly less frequently to secondary care, but the latter referred more frequently to radiological examination. Our findings indicate that intervention on high referrers is a potential area for quality improvement, and there is a need to explore the referral decision process itself.

## Background

As in most Western countries, the political goal in Norway is that health care should be allocated according to medical need and at the lowest effective care level. The referral rates of general practitioners (GPs) are an important determinant of secondary care utilization [1]. However, research over the last 30 years has established that referral rates vary considerably between GPs, and that the variation in these rates cannot be explained by patient morbidity alone. Other factors play an important role, such as health care organization, GP characteristics and patients’ wishes [2,3].

Access to secondary care varies considerably between countries. In the United States patients can refer themselves to secondary care. But in countries like Denmark, the Netherlands, the United Kingdom, Spain, Iceland, and Norway, referrals to secondary care are largely controlled by GPs. Primary health care is well developed in Norway; still, only 55% of Norwegian GPs are specialists in family medicine [4], and no speciality is required to practice as a GP. The Norwegian GPs work either as private practitioners, with capitation payment and fee-for-service reimbursements, or salaried, employed by the municipality. The density of GPs is near the average among the OECD countries, and the density of secondary care specialists is above average [5]. The total health expenditure per capita in Norway was 5352 USD PPP (US Dollars Purchasing Power Parity) in 2009, only surpassed by the US [6]. The patient list system, introduced in 2001, comprises 99% of the population. During this same period, the function of the GP as a gatekeeper to secondary care was emphasized. There are no financial incentives related to referrals. Patients are entitled to receive necessary health care from secondary care specialists only if the they are expected to benefit from it, and if the costs are reasonable in relation to the effect of the intervention [7]. However, The Norwegian Patients’ Rights Act also states that the patients have a right to participate in the decision of which available and medically sound methods of examination and treatment they will receive [8]. This creates a dilemma, and many GPs find it difficult to identify with and fill the gatekeeper role [9].

Referrals have different definitions across studies and countries, which constrain the possibilities for comparison. Referrals may be defined as referral to all types of investigations and treatment by secondary care, including radiological examination and laboratory tests, or as parts thereof. Moreover, referral rates are calculated with different denominators, population (list size) or consultations. Our rates are only compared to studies using consultations as the denominator, and, if not stated otherwise, the nominator containing the sum of hospital outpatient clinics, private practitioner secondary care specialists and hospital admissions. In 1983 Rutle reported a mean referral rate of 8% (8 per 100 consultations) in a Norwegian general practice. About a decade later Fleming et al reported a Norwegian referral rate of 8.2%, the highest rate among the participating countries in his European study, in which the Danish referral rate was 6.6% and the British one was 4.7% [10]. In 1997 Roland et al reported a referral rate range to National Health Service secondary care in Scotland of 1.7 to 7.8% [11]. In Denmark, Moth et al reported a 2009 referral rate of 8.4% to hospital outpatient clinics, hospital admissions and private secondary care specialists [12].

In most OECD countries there is a rising concern about the increasing health care costs. There is also a continuing worry about the range of referral rates which raises the concern of both over- and under-use of secondary care [13]. Especially, since Norway has high health care expenditures, it is important to monitor and update the knowledge on referral rates and range among GPs and to compare them with past and more recent surveys. Likewise, too little is known about factors affecting the decision to refer. Thus, the main objective of this study was to assess the GPs’ referral rate to secondary care in Norway, any associations between the referral decision and patient, GP, health care characteristics and who initiated the referral issue in the consultations. The latter has to our knowledge not been much studied before.

## Methods

### Recruitment and data collection

Of all 476 GPs (lists) in Northern Norway 88 GPs were excluded, due to electronic patient records (EPR) incompatibility with our electronic questionnaire (n= 44), vacancy (n=35), the two practices housing 3 GPs participating in piloting (n=8), and finally one GP practicing without EPR. Power calculation indicated a need for some 2500 consultation in each subgroup to detect a 25% difference in referrals rates, alpha 0.05 and beta 0.8. With an expected response rate of 50%, a random sample of 104, of the 388 eligible GPs, was invited to participate in the present study**.** Forty-six accepted to participate after four reminders, and 44 GPs in 22 practices completed the study. This gives a response rate of 42% (44/104).

Information collected from a background questionnaire included the GPs’ age, gender, specialization in family medicine, type of practice, number of GPs in the practice, country where medical degree was obtained, list size, mean years with current list population, and days per week in clinical practice. From Northern Norway Regional Health Authority (Helse Nord RHF: http://www.helse-nord.no/) we got information on primary and secondary care institutions and radiological services in the municipalities. The population size of the municipalities and travel time for patients to the nearest hospital were provided by Statistics Norway.

Referral data from 100 consecutive consultations per GP were collected between November 2008 and September 2010. The GPs spent between 7 and 19 days to complete the electronic questionnaires, which popped up after the GP closed the EPR for each patient. In the questionnaires, the GP recorded whether the issue of referral was introduced during the consultation, who introduced the issue, and if the patient was referred to secondary care (hospital outpatient services, private secondary care specialists, hospital admissions, rural psychiatric services, and other specialists) and/or radiological examination.

### Statistical analyses

All analyses were performed with Stata 12.0. Referrals to secondary care and radiological examination were analy sed separately. Logistic regression analysis was first performed with a multilevel method, with possible clustering on GP level. Multilevel analysis was only significantly better than naive analysis for referrals to radiological examination. Analyses of referral to secondary care were therefore performed with naive logistic multivariable regression analysis. Due to a relatively low number of GPs and therefore reduced power, we included variables with a significance level less than 0.15 in the final model in each regression analysis, after testing that removing the variables did not lead to a poorer-fitting model. We always adjusted for the gender and age of both patients and GPs.

The Data Protection Official for Research approved the survey.

## Results

The study comprised 4350 consultations. The patients’ mean age was 50 years, and 56.1% of patients were female. Eighteen per cent of the patients lived in towns with more than 40 000 inhabitants, and about half lived in municipalities with less than 5000 inhabitants. Twenty-eight per cent of patients had less than 30 minutes of travel time to reach the nearest hospital, and about half had more than 90 minutes of travel time, data not shown.

Selected characteristics of the GP sample, of the non-responders, and of the GP population in Northern Norway are given in Table 1. Our GP sample did not differ substantially from the GP population, except for a slightly lower percentage of GPs in private practice and a slightly higher percentage with medical degrees from Norwegian universities. Compared to non-responders, the responders were younger, more were specialists in family medicine and practiced closer to hospitals in larger municipalities. More responders were private practitioners and more had their medical degree from Norway. However, information of the last two characteristics was only available for about 80% of the non-responders.

**Table 1:** **Characteristics of the responders**^**1**^

**Characteristics**	**Responders**			**Non-responders**	**Population of GPs in Northern Norway**
	Male	Female	Total	Total	N=476^2^
	GP	GP	sample		
	n=30	n=14	n=44	n=60	
					
Mean age, year	45.2	44.6	45.1^3^	50.3^3^	45.0^**4**^
Gender, %	68.2	31.8		31.7% female	36.3 % female^**4**^
Specialists in family medicine, %	50.0	50.0	50.0	40.0	46.5^**4**^
Private practice, %	63.3	57.1	61.4	51.1^5^	76.5^**4**^
Mean number of GPs in practices	4.5	4.2	4.4	3.6	3.8
Solo practice^6^, %	10.0	14.3	11.4	8.3	11.1^**4**^
Medical degree from Norway, %	70.0	71.4	70.5	46.9^5^	62.1^**4**^
Mean list size, persons	897	902	899	910	961^7^
Mean years with current list population (median)	8.6	8.6	8.6 (5.5)		
Mean number of days per week in clinical practice	3.9	3.8	3.8		
Mean (median) municipality population in the GP’s workplace	11683	26042	16257	10791	5291
					(2209)
Mean travel time to nearest hospital, minutes	89	70	83	95	

^1^Comparing the responders with non-responders and with the population of GPs in Northern Norway.

^2^All GP positions in Northern Norway, autumn 2008.

^3^ p=0.02.

^4^ Statistics of Physicians in Northern Norway on December 22^nd^ 2008 based on members of Norwegian medical association below 70 years by Anders Taraldset, Chief of Statistics in the Norwegian Medical Association, Personal communication March 30th 2011.

^5^ Statistics of Physicians in Northern Norway on July 1st 2009 based on members of Norwegian medical association below 70 years by Anders Taraldset, Chief of Statistics in the Norwegian Medical Association, Personal communication December 19th 2012,

Private practice, %: n=47 and Medical degree from Norway, %: n=49.

^6^ Only one GP in the practice.

^7^ Norwegian Directory of Health: “Data from GP service” (Data fra allmennlegetjenesten), page 18, rapport IS-1808, October 28^th^ 2010 http://www.helsedirektoratet.no/publikasjoner/arsrapport-2009-prosjekt-sykestuefinansiering/Publikasjoner/data%20fra%20almennlegetjenesten.pdf.

### Referral to secondary care and radiological examination

Of 4350 patients, 689 were referred to somatic secondary care and/or radiological examination, 45 to psychiatric secondary care and 1 patient to both types of care. 90% of referrals to somatic care were directed to outpatient services; 79% to hospital outpatient services and 21% to private practising secondary specialists. Of all 735 referrals about a quarter were to radiological examination, data not shown.

The distribution of age and gender among patients referred to somatic secondary care and to radiological examination was quite similar. But the mean age of patients referred to psychiatric secondary care was significantly lower than the age of patients referred to somatic secondary care (36.5 years (95% CI 30.8 - 42.2) versus 50.4 years (95% CI 48.8 - 52.0), data not shown.

### Referral to secondary care

The mean referral rate to secondary care was 13.7% (13.7 per 100 consultations) with a striking range of 4.0% to 28.0% among the GPs (Table 2). The referral rate was highest for patients aged 10-29 years, and decreased slightly with age. Female GPs referred significantly more frequently than male GPs (16.0% versus 12.6%, adjusted odds ratio, AOR, 1.25), specialists in family medicine referred less frequently than their counterparts (12.5% versus 14.9%, AOR 0.76), and salaried GPs referred more frequently than private practitioners (16.2% versus 12.1%, AOR 1.36). In multivariable analysis the latter difference only applied to male GPs (AOR 1.58), data not shown. The likelihood of referral tended to be higher among GPs aged 35-49 years compared to younger and older colleagues, and lower if the travel time for patients to the nearest hospital was more than 90 minutes.

**Table 2:** **GPs’ referral rates to secondary care, N=4350 consultations**^**1**^

		**#GPs**	**Mean referral rates per 100 consultations**	**(n / N)**	**Adjusted**	**95% CI**
					**odds ratio**	
			**% (median)**			
Total mean (median) referral rate			13.7 (13.0)	(595/4350)		
			Range: 4.0 - 28.0			
Variables						
						
Patients’ gender	Male, 0		13.0	(248/1911)		
	Female, 1		14.2	(347/2439)	1.05	(0.88 - 1.27)
						
Patients’ age	0-9 years		8.0	(19/238)	0.43^2^	(0.26 - 0.73)
	10-29 years		16.4	(104/636)	1	
	30-49 years		15.4	(180/1166)	0.90	(0.69 - 1.18)
	50-69 years		13.3	(176/1321)	0.77^3^	(0.59 - 1.0009)
	>=70 years		11.7	(116/989)	0.64^2^	(0.48 - 0.86)
						
GPs’ gender	Male, 0	30	12.6 (12)	(376/2977)		
	Female, 1	14	16.0 (13)	(219/1373)	1.25^2^	(1.03 - 1.52)
						
GPs’ age	< 35 years	12	13.0 (11)	(152/1174)	0.70^2^	(0.55 - 0.91)
	35 – 49 years	17	16.1 (15)	(270/1676)	1	
	>= 50 years	15	11.5 (12)	(173/1500)	0.83	(0.65 - 1.07)
						
Specialists in family medicine	No, 0	22	14.9 (15)	(321/2149)		
	Yes, 1	22	12.5 (12)	(274/2201)	0.76^2^	(0.60 - 0.96)
						
Solo practice	No, 0	39	13.3 (12)	(510/3850)		
	Yes, 1	5	17.0 (15)	(85/500)	1.30	(0.98 - 1.71)
						
Practice type	Private practice, 0	27	12.1 (12)	(324/2675)		
	Salaried, 1	17	16.2 (16)	(271/1675)	1.36^2^	(1.11 - 1.66)
						
Travel time to nearest hospital	<30 minutes	12	13.0 (12)	(156/1201)	0.94	(0.70 - 1.25)
	30 - 89 minutes	12	16.7 (15)	(192/1148)	1	
	>= 90 minutes	20	12.3 (12)	(247/2001)	0.80^4^	(0.64 - 1.02)
						
Country where medical degree was obtained	Abroad=0	13	14.8 (14)	(184/1247)	Not included in model	
	Norway=1	31	13.3 (12)	(411/3103)		

^1^Mean referral rates and probability of referral by various variables analyzed by naive multivariable logistic regression.

^2^ p<0.05.

^3^ p=0.051.

^4^ p=0.068.

### Referral to radiological examination

The mean referral rate to radiological examination was 4.2%, ranging from 0.0% to 12.9% among the GPs (Table 3). Patients aged 30-49 years were most frequently referred. Specialists in family medicine referred significantly more frequently than GPs who were not specialized in family medicine (5.1% versus 3.2%, AOR 1.93), salaried GPs more frequently than private practitioners (5.2% versus 3.5%, AOR 2.00), and those with their medical degree from Norway more frequently than their counterparts (4.6% versus 3.1%, AOR 1.73).

**Table 3:** **GPs’ referral rates to radiological examination, N=4350 consultations**^**1**^

		**#GPs**	**Mean referral rates,**	**(n / N)**	**Adjusted**	**95% CI**
			**per 100 consultations**		**odds ratio**	
			**% (median)**			
Total mean (median) referral rate			4.2 (4.0)	(181/4350)		
			Range: 0.0-12.9			
Variables						
						
Patients’ gender	Male, 0		4.1	(79/1911)		
	Female, 1		4.2	(102/2439)	0.96	(0.70 - 1.30)
						
Patients’ age	0-9 years		2.1	(5/238)	0.40^2^	(0.16 - 1.02)
	10-29 years		3.0	(19/636)	0.56^3^	(0.33 - 0.95)
	30-49 years		5.2	(61/1166)	1	
	50-69 years		4.4	(58/1321)	0.83	(0.57 - 1.20)
	>=70 years		3.8	(38/989)	0.70	(0.46- 1.07)
						
GPs’ gender	Male, 0	30	4.0 (4.0)	(120/2977)		
	Female, 1	14	4.4 (3.0)	(61/1373)	0.98	(0.61- 1.57)
						
GPs’ age	< 35 years	12	2.9 (2.0)	(34/1174)	0.96	(0.49 - 1.86)
	35 – 49 years	17	5.1 (4.1)	(86/1676)	1	
	>= 50 years	15	4.1 (3.0)	(61/1500)	0.72	(0.41- 1.26)
						
Specialists in family medicine	No, 0	22	3.2 (3.0)	(68/2149)		
	Yes, 1	22	5.1 (4.0)	(113/2201)	1.93^3^	(1.05 - 3.55)
						
Solo practice	No, 0	39	4.3 (4.0)	(164/3850)	Not included in model	
	Yes, 1	5	3.4 (4.0)	(17/500)		
						
Practice type	Private practice, 0	27	3.5 (2.0)	(94/2675)		
	Salaried, 1	17	5.2 (5.0)	(87/1675)	2.00^3^	(1.21 - 3.31)
						
Travel time to nearest hospital	<30 minutes	12	4.4 (2.0)	(53/1201)	0.99	(0.52 - 1.86)
	30 - 89 minutes	12	5.4 (5.0)	(62/1148)	1	
	>= 90 minutes	20	3.3 (3.0)	(66/2001)	0.66	(0.39 - 1.12)
						
Country where medical degree was obtained	Abroad=0	13	3.1 (3.0)	(39/1247)		
	Norway=1	31	4.6 (4.0)	(142/3103)	1.73^3^	(1.03 – 2.89)

^1^Mean referral rates and probability of referral by various variables analyzed by multilevel multivariable logistic regression.

^2^ p=0.054.

^3^ p<0.05.

### Introducing the issue of referral in the consultations and corresponding referral rates

The issue of referral was introduced in 23% of the consultations (Table 4); introduced by the GPs in 70.6% (707/1001) of these, and by the patients in 29.4% (294/1001), but resulted in a referral rate that was surprisingly very much the same (59.4% and 59.5%, respectively).

**Table 4:** **Introducing the referral issue in the consultations and corresponding mean referral rates**^**1**^

**GP**	**Lowest quartile,**		**Highest quartile,**		**All GPs**	
**strata**						
	**referral rate**		**referral rate**			
	**<10% ,**		**>=16%,**			
	**n = 1102**		**n = 1047**		**n = 4350**	
	**consultations**		**consultations**		**consultations**	
	**n = 11 GPs**		**n = 11 GPs**		**n = 44 GPs**	
						
	Referral	Referral	Referral	Referral	Referral	Referral
	issue	rate	issue	rate	issue	rate
	introduced		introduced		introduced	
	%	%	%	%	%	%
	(95% CI)	(95% CI)	(95% CI)	(95% CI)	(95% CI)	(95% CI)
	(n/N)	(n/N)	(n/N)	(n/N)	(n/N)	(n/N)
Referral rate,						
%		7.8		20.9		13.7
(95% CI)		(6.2 – 9.4)		(18.5 –23.4)		(12.7–1 4.7)
(n/N)		(86/1102)		(219/1047)		(595/4350)
						
Referral	18.0	43.4	31.3	66.8	23.0	59.4
issue	(15.7–20.2)	(36.5–50.4)	(28.5- 34.1)	(61.7–71.9)	(21.8 - 24.3)	(56.4 - 62.5)
introduced	(198/1102)	(86/198)	(328/1047)	(219/328)	(1001/4350)	(595/1001)
						
Referral						
issue of	26.3	51.9	29.0	66.3	29.4	59.5
introduced	(20.3 - 33.0)	(38.1–65.7)	(24.1 - 34.2)	(56.7 –75.9)	(26.6 – 32.3)	(53.9 - 65.2)
by patient	(52/198)	(27/52)	(95/328)	(63/95)	(294/1001)	(175/294)
						
Referral						
issue	73.7	40.4	71.0	67.0	70.6	59.4
introduced	(67.0 - 79.7)	(32.4–48.5)	(65.8 - 75.9)	(60.9 –73.4)	(67.8 - 73.5)	(55.8 - 63.0)
by GP	(146/198)	(59/146)	(233/328)	(156/233)	(707/1001)	(420/707)

^1^Comparing strata of GPs, by referral rate.

The referral issue was introduced in 31.3% of the consultations among high referrers (top quartile), and 66.8% of these patients were referred (Table 4). Among low referrers (lowest quartile), the referral issue was introduced significantly less frequently, in only 18.0%, of which only 43.4% were actually referred.

When the high referrers introduced the issue themselves, they referred significantly more frequently than the low referrer, 67% versus 40.4%. When the patients introduced the issue, the difference between the subsequent referral rates among the high and low referrers tended to be smaller.

In sub-analyses in our study we found that the issue of referral was introduced to about the same extent in consultations with female and male GPs, 23.96% (95% CI 21.7 – 26.2) and 22.6% (95% CI 21.1 – 24.1), but the female GPs referred a significantly larger proportion of patients than males when the issue of referral was introduced (OR=1.56, 95% CI 1.19- 2.06), especially when the female GPs introduced the issue themselves, (OR=1.7, 95% CI 1.22 – 2.39).

## Discussion

### Summary of main findings

The main finding of the present study was a high overall referral rate compared to a similar study from a compa rable country [12] and a substantial increase compared to past studies in Norway [10,14]. We also observed a striking range in referral rates among the GPs. Male GPs referred significantly less frequently to secondary care than female GPs. Specialists in family medicine referred less frequently to secondary care, but more often to radiological examination. The issue of referral was introduced in 23% of the consultations, introduced by the GPs in 70.6% of these, and by the patients in 29.4%, but the following referral rate was very much the same. The high referrers introduced the referral issue significantly more frequently and also referred a significantly larger proportion when the issue was introduced.

### Strengths and limitations of the study

A considerable strength in our survey is that the questionnaires, of the consultations studied, mandatorily popped up and were consecutively filled out immediately after each consultation, giving a sample of representative patients. Furthermore, the GP sample was randomly drawn and altogether the referral data was collected through all parts of the year.

However, the response rate of 42% raises the concerns of selection bias. Others have found that non-responder GPs tend to be older and less likely to possess a postgraduate qualification [15]. Our responders were indeed younger than the non-responders and were more often specialists in family medicine (Table 1). Furthermore, more responders worked in private practice and were more often medically educated in Norway. It is difficult to assess the overall impact of non-response bias. But, assuming that the referral practice of the non-responders was similar to that of the responders, increasing the response rate might lower the referral rate related to increasing GP age, especially of those above 50 years. On the other hand, increasing the response rate might also increase the referral rate related to a falling percentage of specialists in family medicine, non-salaried GPs and GPs with medical degree from Norway. In a set of hospital data, which comprised all referrals from all GPs to any hospital in Northern Norway (hospital outpatient clinics only) between 2008 and 2011, our responders referred 23.4% of their list population per year compared to 25.6% among non-responders, (personal communication with Center of Clinical Documentation and Evaluation (SKDE), Northern Norway Regional Health Authority of May 2011). This might indicate that the observed referral rates in our study more probably represent an underestimation than an overestimation of referrals. Additionally, the total secondary care utilization in Northern Norway does not differ substantially from the rest of the country according to a Norwegian report [16]. We therefore believe that our study is fairly representative of the GP referral practice in Norway.

### Comparison with existing literature

The referral rate in Norway was reported to be 8.0% in 1983 [14] and 8.2% in 1993 [10]. In the same time period the referral rate in Finland was 4.5% to out- and inpatients services [17], and the British one was 4.7% [10]. In 2009 the referral rate in Denmark was reported to be 8.4% [12]. There may be problems with comparing referral rates between countries because the consultation practice may differ, i.e. the consultation rate per day may differ. Still, our referral rate of 13.7% to secondary care was higher than anticipated [2]. This increase in referral rate in Norway of 5.5 percentage points from 1993, represents a secondary care work load increase of about 67% and may indicate a less restrictive GP gatekeeper function [9]. The indicated work load increase is supported by the fact that the number of outpatient consultations in Norway has increased by more than 60% between 2002 and 2011 [18]. In the United States the reported probability that an ambulatory visit to a phy sician would result in a referral to another physician, increased by 94%, from 4.8% in 1999 to 9.3% 2009 [19]. Also Moth et al documented a 36% rise in the referral rate to secondary care in Denmark (radiology and laboratory excluded: from 6.2% in 1993 to 8.4% in 2009) [12].

The GP consultation rate has increased somewhat over time, with a rise of around 5.4% between 2006 and 2009 [20]. The increased referral rate may partly be a result of care becoming more complex, thereby requiring more care by secondary care specialists. In addition, the diagnostic and therapeutic options have expanded, and are applied to patients with either more advanced disease, or who are older. Also, patient demand and consumerism are contributing factors. An increase in the referral rate may have unwanted consequences for the patients. Unnecessarily referred patients may be subjected to unnecessary and dangerous diagnostic and therapeutic procedures [21]. In addition, an increasing referral rate contributes to longer patient waiting lists which may cause poorer health outcomes for seriously ill patients [22]. Increasing referral rates also increase the economic burden of health care on society.

The wide range of referral rates between GPs has been well documented, but our reported seven-fold increase is among the highest ever reported [2,11]. The variation in referral rates seems to be stable over time and independent of case mix [23]. Sullivan et al reported that morbidity only explained 30.4% of the total variation in referral rates, and patients’ age and sex explained 5.3% of the total variation [3]. Thus, non-medical factors must also contribute to the referral rate range. In our study, the relative proportion of referrals was about the same whether the patient or the GP initiated the issue of referral. We had anticipated that the referral rate would be higher when GPs initiated the issue of referral, and we therefore think our result indicates a stronger responsiveness to the patient’s requests on the part of the GP, resulting in difficulties or reluctance to make rationing decisions. The issue of referral was introduced more frequently in consultations with high referrers, and also resulted more often in referrals. This may be due to higher professional insecurity and/or higher responsiveness to patient demands. The variability in referral rate challenges the basic principle of equal access to health care, as patients with low referrers as their GPs encounter a different health care system than those attending high referring ones.

In the present study the referral rate of female GPs to secondary care was 25% higher than that of male GPs. This is consistent with gender differences found in other studies. Vehvilainen et al found that female GPs referred 22% more than male GPs (female rate 5.48% versus 4.50% among males) [24]. One explanation for this difference may be gender differences in risk tolerance. Indeed, GPs with lower risk tolerance are reported to refer more [25,26], and a significantly lower risk-taking behavior has been found in females in a meta-analysis [27]. In sub-analyses in our study we found that female GPs referred a significantly larger proportion of patients than males when the issue of referral was introduced, especially when the GPs initiated the issue. This may indicate a female tendency to stronger acquiescence and an attempt to reduce uncertainty when choosing to refer. As the percentage of women is increasing among doctors, the reported gender difference could have major implications for patients and society. The literature, however, reveals conflicting evidence regarding whether women practice medicine differently than men [28].

We found that specialists in family medicine referred less to secondary care, which has also been reported by others [24]. However, these specialists referred more frequently to radiological examination. May be specialists in family medicine perform more of the diagnostic process themselves instead of referring them to secondary care, or they start investigating the patient while waiting for secondary specialist consultation.

We were surprised to find a significant difference in the probability of referral between salaried male GPs and those in private practice, but no difference among female GPs in these types of practices in multivariable analyses. All private practitioners had significantly more consultations per day than salaried GPs (mean 10.4 per day, 95% CI 9.6-11.3, versus 7.8, 95% CI 6.6-8.9, respectively). It may be that salaried GPs have fewer, but longer consultations per week and refer more frequently per consultation, but not more frequently per list population per year. Our finding is somewhat in line with Fleming, who in 1993 reported that the low referring doctors undertook a high number of consultations compared to high referring ones [10].

The relatively low number of GPs in our survey and lack of power may give concern of type II errors. We will therefore also display non-significant results. Proximity to specialist is reported to increase referral rates [2]. We found that the probability of being referred were 20% less for patients with the longest travel time (Table 2).

### Implications for general practice and future research

We found a high referral rate and a very large range. Although our study does not consider the appropriateness of the referral rates, the lower referral rate of specialists in family medicine indicates that the overall rate may be lowered. Future research should focus on exploring the effectiveness of quality improvement measures and include strategies to both reduce the level and range of referral rates. Our results from contrasting high and low referrers may indicate that intervention on high referrers is a potential area for quality improvement. In a meta-analysis concerning interventions to influence referral patterns, Akbari et al ascertained that few rigorous evaluations have been carried out [29]. Active local educational interventions involving secondary care specialists and structured referral sheets were the only interventions shown to have an impact on referral rates. The effects of ‘in-house’ second opinion and other intermediate primary care-based alternatives to outpatient referral appeared promising.

GP’s gender, specialization in family medicine and type of practice have an important impact on referral rate. The frequency of introducing the issue of referral in the consultation and the corresponding referral rates are different in high and low referrers. This needs to be explored more to shed light on the communication skills of GPs, and to understand how gender, patients’ wishes and other issues relevant to equality and diversity affect the way GPs practice medicine [28,30,31].

In Norway a Coordination Reform [32] began its implementation in January 2012. There is a need to control the demand for secondary health care at the primary health care level, and a system of copayment for secondary care use from the municipality, is introduced. One goal of the reform is to prioritize development of new services in the municipalities and transfer patients from secondary to primary health care when necessary. The high referral rates found in our study call into question the viability of the government’s goal with the reform.

## Conclusions

The main finding of the present study was a high overall referral rate, and a striking range among the GPs. Male GPs and specialists in family medicine referred significantly less frequently to secondary care, but the latter referred more frequently to radiological examination. Our findings indicate that intervention on high referrers is a potential area for quality improvement and there is a need to explore the referral decision process itself.

## Abbreviations

GP: General practitioner; AOR: Adjusted odds ratio; EPR: Electronic Patient Record; 95% CI: 95% confidence interval; OR: Odds ratio.

## Competing interests

All authors declare no financial and non-financial interest.

## Authors’ contribution

UR participated in the design of the study, did the acquisition of the data, performed the statistical analysis, and drafted and critically revised the manuscript. NF participated in the design of the study, helped in the statistical analyses, and critically revised the manuscript. TSD participated in the design of the study, helped in the statistical analyses and critically revised the manuscript. TH participated in the design of the study and critically revised the manuscript. OHF conceived of the study, participated in the design of the study, helped in the statistical analyses, and drafted and critically revised the manuscript. All authors have approved the final version of the manuscript.

## Pre-publication history

The pre-publication history for this paper can be accessed here:

http://www.biomedcentral.com/1472-6963/13/147/prepub

## References

[B1] Forde OH Breidablik HJ Ogar P: **[Do differences in referral rates threaten the goal of equity in health care?]** Tidsskr Nor Laegeforen 2011 131 1878 1881 21984292 10.4045/tidsskr.10.1450

[B2] O’Donnell CA Variation in GP referral rates: what can we learn from the literature? Fam Pract 2000 17 462 471 10.1093/fampra/17.6.462 11120716

[B3] Sullivan CO Omar RZ Ambler G Majeed A Case-mix and variation in specialist referrals in general practice Br J Gen Pract 2005 55 529 533 16004738 PMC1472770

[B4] Statistics on GPs - FAQs (Legeforeningens fastlegestatistikk - svar på ofte stilte spørsmål 2012 Oslo: The Norwegian Medical Association http://legeforeningen.no/Emner/Andre-emner/Legestatistikk/Yrkesaktive-leger-i-Norge/Legeforeningens-fastlegestatistikk---artikkel/ 6-12-2012 Ref Type: Online Source

[B5] OECD Health Data 2009 - comparing health statistics across OECD countries PAC/COM/NEWS 2009 http://search.oecd.org/officialdocuments/displaydocumentpdf/?cote=PAC/COM/NEWS(2009)17&docLanguage=En. 2009. Paris, France. 13-6-2012 Ref Type: Online Source

[B6] Health at a Glance 2011: OECD Indicators, Figure 7.1.1 Total health expenditure per capita, public and private 2009 or nearest year). 2012. 6-12-2012 Ref Type: Online Source

[B7] The Act of 2 July 1999 No. 63 relating to Patients’ Rights (the Patients’ Rights Act), section 2-1 1999 Oslo, Norway http://lovdata.no/info/lawdata.html 13-10-2011 Ref Type: Online Source

[B8] The Act of 2 July 1999 No. 63 relating to Patients’ Rights (the Patients’ Rights Act), section 3-1 1999 Oslo, Norway http://lovdata.no/info/lawdata.html 13-10-2011 Ref Type: Online Source

[B9] Carlsen B Norheim OF “Saying no is no easy matter” a qualitative study of competing concerns in rationing decisions in general practice BMC Health Serv Res 2005 5 70 10.1186/1472-6963-5-70 16281967 PMC1291367

[B10] Fleming DM The European study referrals from primary to secondary care 1993 Mastricht: University of Limburg

[B11] Roland M Grimshaw J Grol R Shanks D Johnson A Russell I Do general practitioner attitudes and characteristics of their practices explain patterns of specialist referral? Eur J Gen Pract 1997 3 143 147 10.3109/13814789709160350

[B12] Moth G Olesen F Vedsted P Reasons for encounter and disease patterns in Danish primary care: Changes over 16 years Scand J Prim Health Care 2012 30 70 75 10.3109/02813432.2012.679230 22643150 PMC3378007

[B13] Raleigh V Tian Y Goodwin N Dixon A Thompson J Millett C General practice in London: Supporting improvements in quality 2012 The King’s Fund. 11-12-2012 Ref Type: Online Source

[B14] Rutle O Pasienten fram i lyset: analyse av legekontaktar i primærhelsetenesta, 1/83 edn 1983 Oslo: Gruppe for helsetjenesteforskning

[B15] Stocks N Gunnell D What are the characteristics of general practitioners who routinely do not return postal questionnaires: a cross sectional study J Epidemiol Community Health 2000 54 940 941 10.1136/jech.54.12.940 11076993 PMC1731599

[B16] Kalseth B SAMDATA Sektorrapport for somatisk spesialisthelsetjeneste 2008 [http://www.sintef.no/project/Samdata/Sektorrapport%20somatikk%202008/SAMDATA_Sektorrapport_somatikk-kapittel_5.pdf]. 2009. SINTEF Teknologi og samfunn, Helsetjenesteforskning. 23-5-2012 Ref Type: Online Source

[B17] Haikio JP Linden K Kvist M Outcomes of referrals from general practice Scand J Prim Health Care 1995 13 287 293 10.3109/02813439508996777 8693214

[B18] The Norwegian Directorate of Health Aktivitetsdata presentert i rapportgenerator, opphold fordelt på oppholdstyper 2012 http://cognos.shdir.no/cognos/cgi-bin/ppdscgi.exe?BZ=1AAAA5DClZOoABEwU6VFChpEwa_iksZOGThk6JoRE6VEGBgwSMftgxGyDBAmSfTW7ZHbn2Nw6M0trkQjChk3ZJsCgkwdOGbl~3sCBg_YNGzIlYOSURmLKoAnjhgybNG7OzJlbe2QvSlufQ5q32tGx4OyaAbOvTMw_sa9i0lVO7SWTaHh9hENMLzNWW_uTtgf8Aw== 2012. 21-6-Ref Type: Online Source

[B19] Barnett ML Song Z Landon BE Trends in physician referrals in the United States, 1999-2009 Arch Intern Med 2012 172 163 170 10.1001/archinternmed.2011.722 22271124 PMC3568395

[B20] 2011. The Norwegian Health Economics Administration (HELFO) Analyserapport, fastleger, legevakt og avtalespesialister, aktivitetsstatistikk 2009 http://www.helfo.no/statistikk/Documents/Analyserapport%20Aktivitetsstatistikk%202009.pdf 29-6-2012 Ref Type: Online Source

[B21] Moynihan R Doust J Henry D Preventing overdiagnosis: how to stop harming the healthy BMJ 2012 344 e3502 10.1136/bmj.e3502 22645185

[B22] Fielden JM Cumming JM Horne JG Devane PA Slack A Gallagher LM Waiting for hip arthroplasty: economic costs and health outcomes J Arthroplasty 2005 20 990 997 10.1016/j.arth.2004.12.060 16376253

[B23] Franks P Zwanziger J Mooney C Sorbero M Variations in primary care physician referral rates Health Serv Res 1999 34 323 329 10199678 PMC1089004

[B24] Vehvilainen AT Kumpusalo EA Voutilainen SO Takala JK Does the doctors’ professional experience reduce referral rates? Evidence from the Finnish referral study Scand J Prim Health Care 1996 14 13 20 10.3109/02813439608997063 8725089

[B25] Ingram JC Calnan MW Greenwood RJ Kemple T Payne S Rossdale M Risk taking in general practice: GP out-of-hours referrals to hospital Br J Gen Pract 2009 59 e16 e24 10.3399/bjgp09X394824 19105912 PMC2605545

[B26] Franks P Williams GC Zwanziger J Mooney C Sorbero M Why do physicians vary so widely in their referral rates? J Gen Intern Med 2000 15 163 168 10.1046/j.1525-1497.2000.04079.x 10718896 PMC1495354

[B27] Byrnes JP Gender Differences in Risk Taking: A Meta-Analysis Psychol Bull 1999 125 3 367 383

[B28] Kilminster S Downes J Gough B Murdoch-Eaton D Roberts T Women in medicine–is there a problem? A literature review of the changing gender composition, structures and occupational cultures in medicine Med Educ 2007 41 39 49 10.1111/j.1365-2929.2006.02645.x 17209891

[B29] Akbari A Mayhew A Al-Alawi MA Grimshaw J Winkens R Glidewell E Interventions to improve outpatient referrals from primary care to secondary care Cochrane Database Syst Rev 2008 4 CD005471 http://onlinelibrary.wiley.com/doi/10.1002/14651858.CD005471.pub2/pdf/standard. Ref Type: Online Source 18843691 10.1002/14651858.CD005471.pub2 PMC4164370

[B30] Little P Importance of patient pressure and perceived pressure and perceived medical need for investigations, referral, and prescribing in primary care: nested observational study BMJ 2004 328 444 10.1136/bmj.38013.644086.7C 14966079 PMC344266

[B31] Carlsen B Aakvik A Norheim OF Variation in practice: a questionnaire survey of how congruence in attitudes between doctors and patients influences referral decisions Med Decis Making 2008 28 262 268 10.1177/0272989X07311751 18349435

[B32] The Norwegian Ministry of Health and Care Services The Coordination Reform 2008 http://www.regjeringen.no/upload/HOD/Samhandling%20engelsk_PDFS.pdf. 2008. 17-2-2012 Ref Type: Online Source

